# Isolation and genetic characterization of a novel recombinant HP-PRRSV strain in Jiangxi Province, China

**DOI:** 10.3389/fvets.2025.1678378

**Published:** 2025-10-06

**Authors:** Chenyi Wang, Ruoming Li, Jiajia Chen, Meiling Zhuo, Qi Peng, Lingbao Kong, Ting Wang

**Affiliations:** ^1^Institute of Pathogenic Microorganism, Jiangxi Agricultural University, Nanchang, China; ^2^Nanchang City Key Laboratory of Animal Virus and Genetic Engineering, Nanchang, China; ^3^College of Bioscience and Engineering, Jiangxi Agricultural University, Nanchang, China

**Keywords:** PRRSV-2, virus isolation, genetic characterization, recombination, JXA1-like

## Abstract

Porcine reproductive and respiratory syndrome virus (PRRSV) is a major pathogen responsible for significant economic losses in the global pig industry, primarily causing reproductive failure in sows and respiratory diseases in piglets. In this study, we isolated a novel PRRSV strain, designated NC2023, from clinical samples collected in 2023 from a pig farm in Jiangxi Province, China. The virus was successfully propagated in Marc-145 cells and demonstrated consistent infectivity in iPAM cells. Comprehensive characterization of NC2023 was performed using RT-qPCR, Western blotting, IFA, electron microscopy, plaque purification, and whole-genome sequencing. Phylogenetic and homology analyses of the complete genome revealed that NC2023 belongs to lineage 8 of the PRRSV-2 subtype and forms an independent branch in the evolutionary tree. The genomic length of NC2023 is 15,321 nucleotides, and recombination analysis indicated it is a recombinant strain with specific genomic regions derived from strains JXA1, JXA1-R, and HUN4. Notably, NC2023 exhibits 15 unique amino acid mutations compared to other recombinant strains, along with a 395-amino-acid frameshift mutation in Nsp2. This study reports the isolation and characterization of an HP-PRRSV recombinant PRRSV-2 strain using Marc-145 and iPAM cells, providing key insights into the genetic variation and evolutionary dynamics of PRRSV. These findings contribute to a deeper understanding of PRRSV molecular epidemiology and have important implications for the development of effective control strategies and vaccines.

## 1 Introduction

Porcine reproductive and respiratory syndrome (PRRS) is an acute and highly contagious infectious disease caused by the PRRS virus (PRRSV). The disease is characterized by adverse reproductive outcomes and respiratory symptoms in pigs (1). PRRSV is classified in the order Nidovirales, family Arteriviridae, and genus *Arterivirus*, is an enveloped, single-stranded positive-sense RNA virus with a diameter of approximately 50–65 nm (2–4). The PRRSV genome is approximately 15 kb in length and contains 11 open reading frames (ORFs): ORF1a, ORF1b, ORF2a, ORF2b, ORF3-7, ORF5a, and ORF1aTF (5). ORF1a, ORF1aTF, and ORF1b account for 80% of the total genome. Due to significant genomic variability, PRRSV isolates are classified into two distinct species: *Betaarterivirus suid 1* (PRRSV-1), predominant in European countries, and *Betaarterivirus suid 2*, (PRRSV-2), widely distributed in American and Asian countries (6). Although these two genotypes exhibit only approximately 60% nucleotide similarity at the genome level, they share identical pathological phenotypes (7, 8).

In China, PRRSV-2 is a major pathogen causing significant economic losses to the swine industry, characterized by high genetic variability and frequent recombination events (9). PRRSV can be categorized into four lineages: 1, 3, 5, and 8 (10, 11). Since its emergence in China, lineage 8 has been dominant, encompassing the classic PRRSV strain (CH-1a-like) and the highly pathogenic PRRSV (HP-PRRSV) strain (12, 13). Lineage 1 strains, collectively referred to as NADC30-like viruses, exhibit extensive recombination with strains from other lineages (14–16). Representative strains include HNyc150, Chsx1401, JL580, FJ1402, FJW05 and TJnh151 (17, 18). Lineage 3, represented by strains GM2 and QYYZ, is associated with late-onset infections (19, 20). Lineage 5 strains, first isolated in 1996, have not caused widespread outbreaks, with the representative strain BJ-4 showing high similarity to VR2332 (19, 21).

In recent years, PRRSV has undergone continuous evolution, with increasing virulence driven by gene recombination and variation (22). The polyprotein encoded by ORF1a is cleaved to produce six non-structural proteins (Nsp1 *α*, Nsp1 *β*, and Nsp2-5). Among these, Nsp2 exhibits the greatest variability within the PRRSV genome (23). Deletions, insertions, or mutations in the Nsp2 are frequently used as a molecular marker to differentiate PRRSV strains (24). For instance, the Nsp2 region of HP-PRRSV features a discontinuous 30-amino-acid deletion, while the QYYZ-like PRRSV has a continuous of 36-amino-acid insertion (25). NADC30-like PRRSV exhibits a discontinuous 131-amino-acid deletion, and NADC34-like PRRSV has a continuous of 100-amino-acid deletion in the Nsp2 region (26, 27). The HP-PRRSV strains, which notably possess discontinuous deletions in the Nsp2 gene, were characterized by high virulence, causing high fever, significant mortality rates, and severe respiratory disease and lung pathology (28, 29). While HP-PRRSV (Lineage 8) was once the predominant strain, NADC30-like (Lineage 1) strains have now become dominant, accounting for up to 70% of prevalent strains based on recent surveillance data (28). A critical evolutionary driver has been frequent genetic recombination, particularly between NADC30-like major patent strains and HP-PRRSV (JXA1-like) minor patent strains, leading to complex recombinant viruses with varied pathogenicity (29, 30). These genetic variations in Nsp2 facilitate the virus’s adaptation to diverse environments and immune pressures, potentially enabling its spread among different host populations and contributing to the emergence of new strains (31). Among the structural proteins of PRRSV, GP5, encoded by the highly variable ORF5 gene, exhibits the greatest degree of variation. GP5 contains a signal peptide and two transmembrane regions and is the major glycoprotein of PRRSV, with strong immunogenicity (32). It harbors multiple epitopes associated with virus neutralization and protection (33). The homology of GP5 between PRRSV-1 and PRRSV-2 ranges from 52 to 57%, while within the same subtype, it ranges from 88 and 99% (32). The high variability of GP5 limits cross-protective immune responses among different genotypes (34), potentially leading to the emergence of unique genetic markers for new variants and reduced vaccine efficacy (35). This complex genetic landscape, marked by high mutation rates and recombination, poses substantial challenges for disease control and vaccination strategies in China.

In this study, a novel PRRSV strain designated NC2023 was isolated from clinical samples collected in Nanchang, Jiangxi Province, China. Whole-genome sequencing and phylogenetic analysis revealed that NC2023 clusters within lineage 8 and shows the closest evolutionary proximity to the JXA1 reference strain. Recombination analysis identified NC2023 as a recombinant strain with genomic regions derived from lineage 8 strains JXA1, JXA1-R, and HUN4. Furthermore, extensive amino acid mutations were identified in the Nsp2 region. These findings provide critical insights into PRRSV genetic diversity and evolutionary mechanisms, enhancing our understanding of strain emergence and vaccine efficacy challenges.

## 2 Materials and methods

### 2.1 Sample collection and processing

Clinical samples positive for PRRSV were obtained from a large-scale pig farm in Jiangxi Province during a PRRS outbreak. Lung tissues from nursery pigs were homogenized in DMEM (Solarbio, Beijing, China) and filtered through a 0.22 μm filter. The filtrate was inoculated onto Marc-145 cells and maintained in an environment with 5% CO_2_. When typical cytopathic effects (CPE) of PRRSV were observed in 70% of the cells, both the cells and the supernatant were collected and stored at −80 °C.

### 2.2 Viral RNA extraction and RT-PCR detection

Total RNA was extracted from the samples using Trizol Reagent. cDNA synthesis was performed using the Hifair® Advance Fast 1st Strand cDNA Synthesis SuperMix for qPCR kit (YEASEN, Shanghai, China) according to the manufacturer’s instructions. Using the representative strain CH-1a (Accession Number: AY032626) as a reference, Primer 5.0 software was employed to design two pairs of primers for the detection of PRRSV and the amplification of the ORF5 gene, respectively, (Supplementary Table S1). The PCR reaction volume was 20 μL with the following amplification conditions: initial denaturation at 94 °C for 5 min, followed by 35 cycles of 94 °C for 30 s, 60 °C for 30 s, and 72 °C for 30 s per 1 kb, and a final extension at 72 °C for 10 min. The products were then analyzed using agarose gel electrophoresis.

### 2.3 Virus isolation and identification

The filtrate from the positive samples was inoculated into Marc-145 cells for virus isolation. The virus was blindly passaged for 3 generations and purified through plaque purification. The newly isolated virus was designated as NC2023. The virus was further confirmed via Western blotting. Marc-145 cells were infected with the virus for 24 h, lysed with RIPA buffer at 4 °C, and then mixed with 5 × loading buffer before being boiled in water for 10 min to fully denature the proteins. The samples were subjected to SDS-PAGE electrophoresis at 80 V for 2 h, followed by protein transfer onto a 0.22 μm PVDF membrane at 300 mA. The membrane was blocked with 5% skimmed milk to prevent non-specific binding. After washing three times with TBST, it was incubated overnight at 4 °C with a PRRSV N antibody (Zoonogen, Beijing, China) diluted 1:2000. Following PBS washing, the membrane was incubated with HRP Goat Anti-Mouse IgG(H&L) (Beyotime, Shanghai China) diluted at 1:5000 for 1 h. Finally, ECL luminescent solution (Proteintech, Wuhan, China) was added, and the image was captured and processed using a gel imaging system.

To detect PRRSV via indirect immunofluorescence assay (IFA), Marc-145 cells infected with the virus for 24 h were fixed with 4% paraformaldehyde (G-Clone, Beijing, China) for 30 min at room temperature. After three washes with PBS, the samples were permeabilized with ice-cold methanol for 10 min. Following another PBS wash, the samples were blocked with 5% BSA for 1 h at room temperature. The PRRSV N antibody, diluted at 1:100, was incubated with samples overnight at 4 °C. After a subsequent PBS wash, Alexa Fluor 488-labeled Goat Anti-Mouse IgG (H + L) antibody (Beyotime, Beijing, China) diluted 1:500, was incubated in the dark for 1 h. Images were captured and processed using an inverted fluorescence microscope.

The virus samples were subjected to further characterization using transmission electron microscopy (TEM). First, the virus was propagated in large quantities and the viral supernatant was subsequently harvested. The virus was then concentrated overnight at 4 °C with 5 × PEG8000 solution. This concentrate was collected via centrifugation at 100,000 × g and fixed at a 1:1 ratio with glutaraldehyde at 4 °C. A 10 μL drop of the sample was applied to a carbon-coated grid, and excess liquid was removed with filter paper. The grids were negatively stained with 10 μL of 2% phosphotungstic acid for 60s. Feynman Biotechnology Tech Co., Ltd. was entrusted to observe the copper grid under an H-7800 electron microscope operating at 80–120 kV and to take photographs.

### 2.4 RT-PCR amplification and PRRSV genome sequencing

The complete genome of PRRSV strain NC2023 was amplified using eight pairs of primers that span the entire viral genome. Based on the Full-gene sequence of JXA1-R (Accession Number: MT163314.1) deposited in GenBank, eight pairs of full-length amplification primers were designed (Supplementary Table S1). Using PrimeSTAR® GXL Premix Fast (Takara, Dalian, China) for PCR, eight fragments of the expected lengths were obtained. The PCR products were purified with the HiPure PCR Pure Mini Kit (Magen, Shanghai, China), and the fragments were cloned into the T vector according to the EZ-TA/Blunt Zero pTOPO II Cloning Kit (Genstar, Beijing, China). Positive transformants were selected and cultured overnight in LB medium containing kanamycin, and the recombinant plasmids were extracted using the Plasmid Extraction Kit (TIANGEN, Beijing, China). The recombinant plasmids were verified by *Eco*RI (Takara, Dalian, China) digestion and sent to Tsingke for sequencing. The eight fragments were assembled into a full-length consecutive sequence using DNAstar 11.0 software and submitted to GenBank with the accession number: PV342160.

### 2.5 Sequence analysis

Phylogenetic analysis and sequence alignments were performed using representative PRRSV genomic sequences obtained from GenBank (Supplementary Table S2). Genetic analyses were conducted on both the ORF5 sequence and the complete genome sequence of NC2023. Multiple sequence alignment was performed using Clustal W in MEGA software, followed by the construction of a phylogenetic tree using the neighbor-joining method with 1,000 bootstrap replicates. The secondary structure of the ORF5 region in NC2023 strain was predicted using SWISS-MODEL and Phyre v2.2, and the resulting data, along with the nucleotide sequence, were imported into ESPript3.0 for comprehensive sequence alignment. Additionally, the antigenic epitopes of the mutated amino acids in the Nsp2 protein of NC2023 were predicted via the IEDB online software, with a threshold value set at 0.5 for epitope identification.

Subsequently, a comprehensive recombination analysis of the genome sequences was conducted using Simplot 3.5.1 software. To enhance the clarity and interpretability of the results, data points with similarity values exceeding 0.50 were extracted and visualized with detailed annotations using R v4.3.3. Additionally, DNAstar-MegAlign was utilized to systematically analyze insertions, deletions, and mutations within both the nucleotide and amino acid sequences of NC2023, in comparison with seven representative reference strains from diverse genetic lineages. Furthermore, following sequence alignment using Clustal W/ Clustal V, the phylogenetic relationships of individual open reading frames (ORFs) and the complete genome were subjected to statistical analysis.

### 2.6 Cellular tropism analysis in iPAM cells

To assess the cellular tropism of NC2023 for immortalized porcine alveolar macrophages (iPAM) cells, the isolated virus was inoculated onto iPAM cells. After 24 h, infected cells were processed for downstream analyses: lysed with RIPA buffer for Western blot, Trizol for RT-PCR, and fixed with 4% PFA for IFA.

## 3 Results

### 3.1 Isolation and identification of the virus

**Figure 1:**
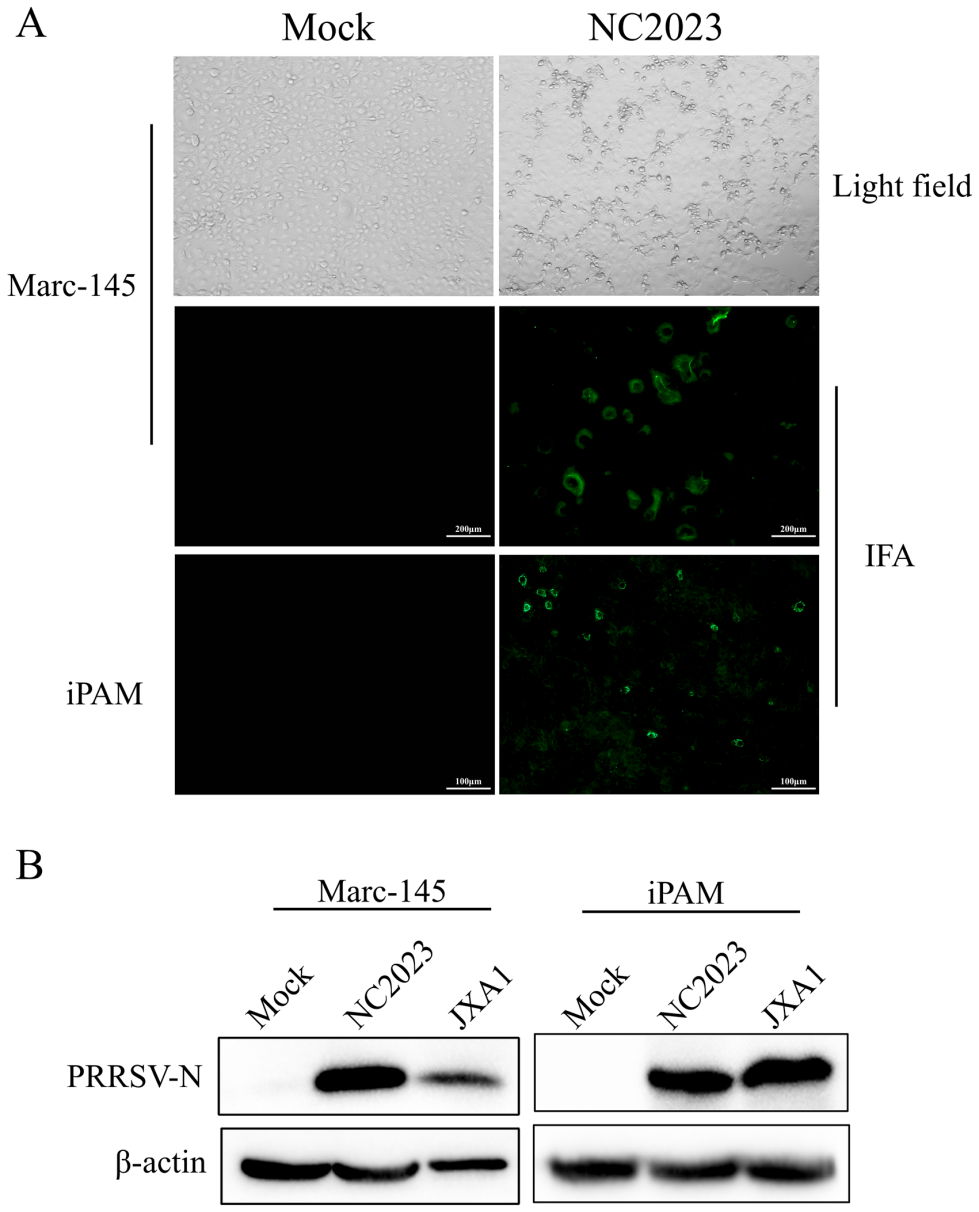
Identification of PRRSV isolates. **(A)** CPE diagrams of blank control of Marc-145 cells (left) and Marc-145 cells infected with PRRSV isolates (right). Result of blank control (left) and indirect immunofluorescence assay of Marc-145/iPAM cells (right). **(B)** Western blot detection of Marc-145 (left)/iPAM cells (right) infected with PRRSV isolates. JXA1: positive control.

**Figure 2:**
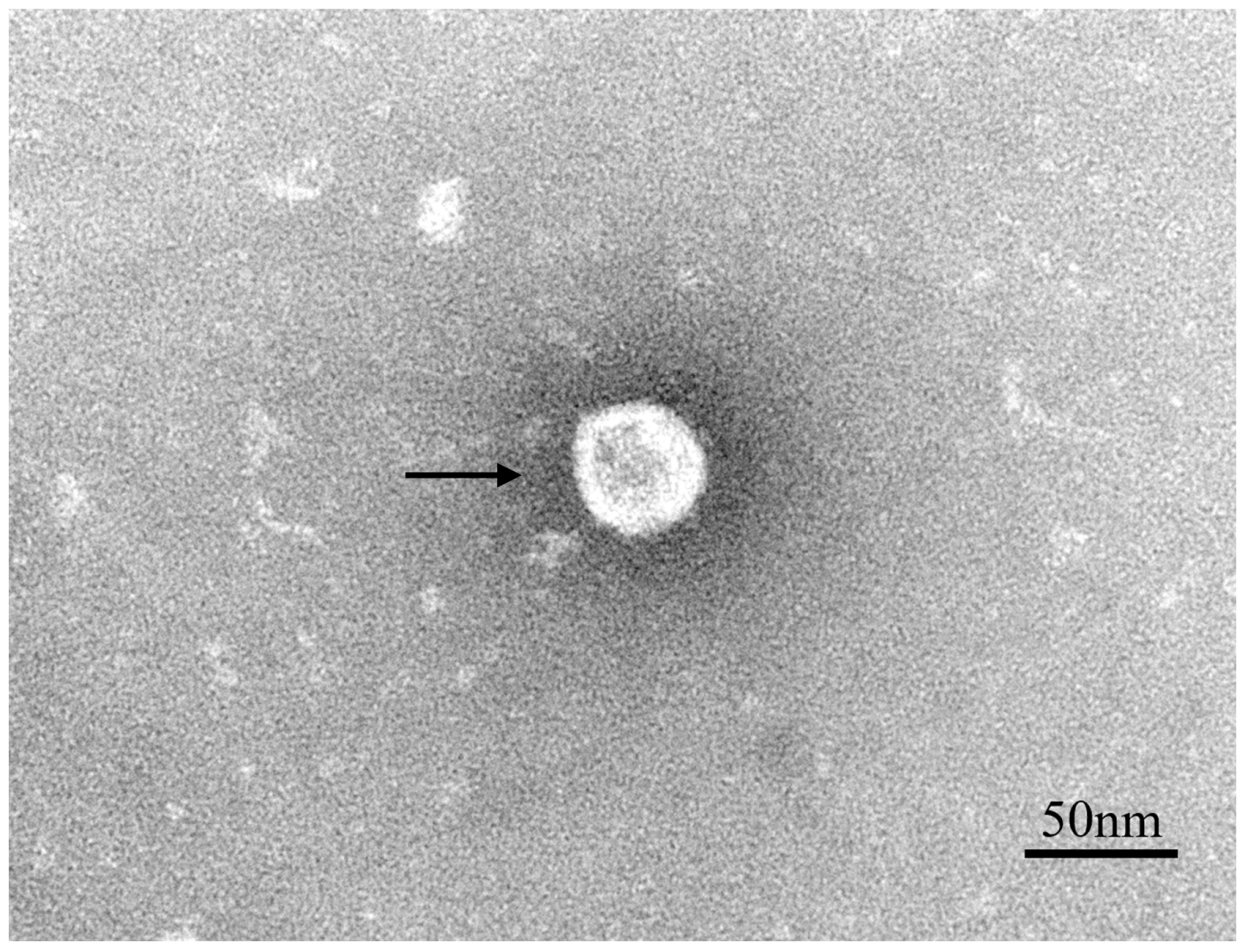
Transmission electron microscope observation of PRRSV virions. The spherical particles marked with black arrows are PRRSV virions.

Lung samples were collected from a suspected PRRSV-infected pig farm in Jiangxi province, China, in 2023. RT-PCR analysis confirmed the lung samples as PRRSV-positive (Supplementary Figure 1A). Homogenized and filtered samples of the infected tissue were inoculated onto Marc-145 cells for virus isolation. Following plaque purification, the novel virus isolate was designated NC2023. This strain was passaged blindly in Marc-145 cells for three times, during which typical PRRSV cytopathic effects were observed but not in the negative control group (Figure 1A). Additionally, immunofluorescence assay (IFA) results demonstrated that Marc-145 and iPAM cells inoculated with NC2023 exhibited specific green fluorescence when probed with anti-PRRSV N antibody, whereas no fluorescence was observed in the negative control group. These findings provide preliminary evidence of successful PRRSV isolation from clinical samples. Further confirmation was obtained through Western blotting (Figure 1B), using uninfected Marc-145 cells as the blank control and cells inoculated with JXA1 as the positive control. The PRRSV N specific protein was detected in cells inoculated with NC2023, confirming the presence of PRRSV in the sample. Transmission electron microscopy revealed the virus particles as circular with a diameter of approximately 40 nm (Figure 2). To investigate the viral replication kinetics over time, the viral titer was quantitatively measured using the TCID50 assay (Supplementary Figure 2). The one-step growth curve revealed that the viral progeny production increased progressively post-infection, reaching a peak titer at 48 h post-infection (h.p.i.).

### 3.2 Genomic characterization

To characterize the complete genome of NC2023, we performed PCR amplification on the viral isolate using eight designed primer pairs for full-genome amplification. Electrophoretic analysis using a 1.5% agarose gel (Supplementary Figure 1D) confirmed that the amplified fragments matched the expected sizes. The sequenced and assembled genome was deposited in the NCBI GenBank under the accession number PV342160. The NC2023 genome spans 15,321 nucleotides (nt), featuring a 189-nt 5′ untranslated region (UTR) and a 150-nt 3’UTR (excluding the poly-A tail), dimensions comparable to previously reported PRRSV-2 strains. Genomic organization analysis revealed eight open reading frames (ORFs) arranged as follows: ORF1a (nt 190–7,612), ORF1b (nt 7,600–11,982), ORF2 (nt 11,984–12,754), ORF3 (nt 12,607–13,371), ORF4 (nt 13,152–13,717), ORF5 (nt 13,699–14,301), ORF6 (nt 14,286–14,810), and ORF7 (nt 14,800–15,171).

### 3.3 Phylogenetic analysis

**Figure 3:**
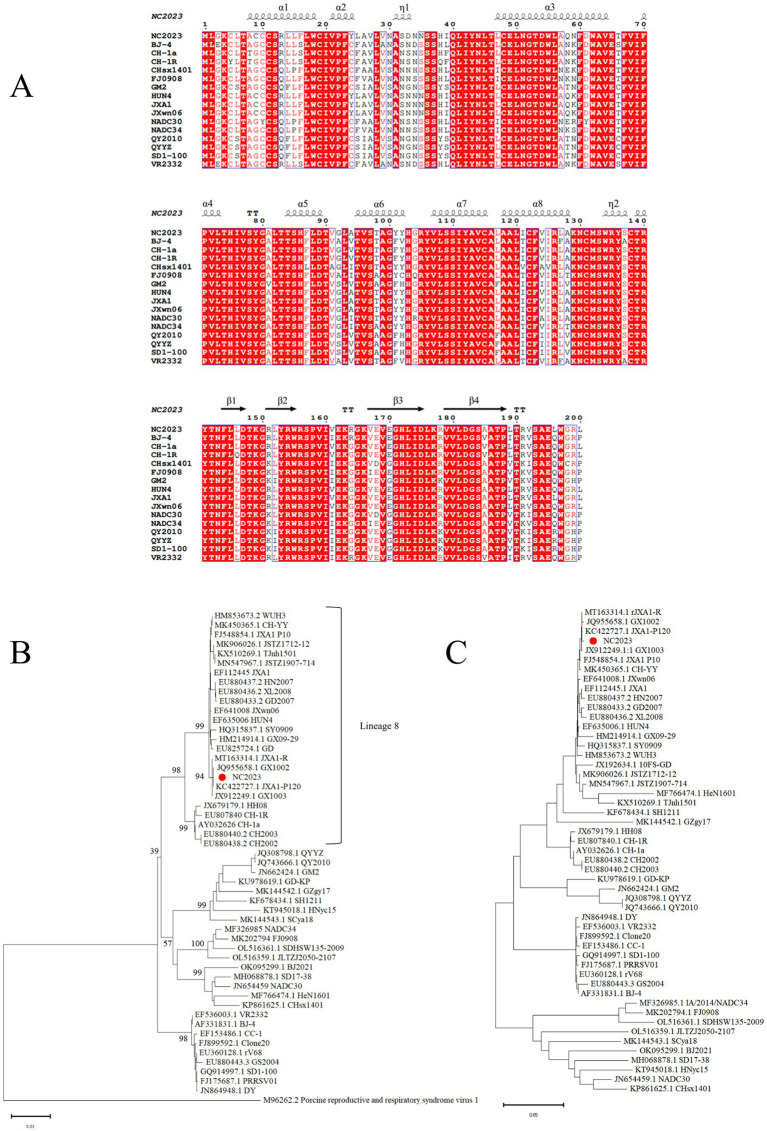
Phylogenetic analysis. **(A)** Results of amino acids alignment of ORF5 of NC2023 with classic PRRSV-2 strains (BJ-4, CH-1a, CH-1R, CHsx1401, FJ0908, GM2, HUN4, JXA1, JXWn06, NADC30, NADC34, QY2010, QYYZ, SD1-100, VR2332). **(B)** Phylogenetic analysis of PRRSV based on ORF5, with the phylogenetic tree constructed using MEGA 11.0, and the red circle representing the NC2023 strain. **(C)** Phylogenetic analysis of PRRSV based on the full genome.

The ORF5 gene of PRRSV is variable and commonly used for phylogenetic analysis. We designed primers to amplify the ORF5 gene, yielding a 600 bp band consistent with the expected size of ORF5 (Supplementary Figures 1B,C). The amino acid sequences and secondary structures of the NC2023 ORF5-encoded protein were compared with those of classical PRRSV-2 subtype strains (Figure 3A), revealing high genetic conservation in both amino acid sequences and spatial structures. To elucidate the evolutionary relationship between NC2023 and reference PRRSV strains, phylogenetic trees were reconstructed using both ORF5 sequences and full-length genomic sequence. ORF5-based phylogenetic analysis positioned NC2023 within the PRRSV-2 lineage, specifically clustering in sublineage 8.3 (Figure 3B). The closest genetic relatives to NC2023 included two regionally prevalent HP-PRRSV variants (GX1002 and GX1003), which share high homology with JXA1 while possessing distinct genetic markers. Notably, the cluster also contained JXA1-P80 (a derivative of JXA1 attenuated through 80 serial passages) and JXA1-R (a genetically modified live-attenuated vaccine strain derived from JXA1). This result is corroborated by the phylogenetic analysis based on complete genome sequences (Figure 3C).

### 3.4 Analysis of sequence insertion and mutation

**Figure 4:**
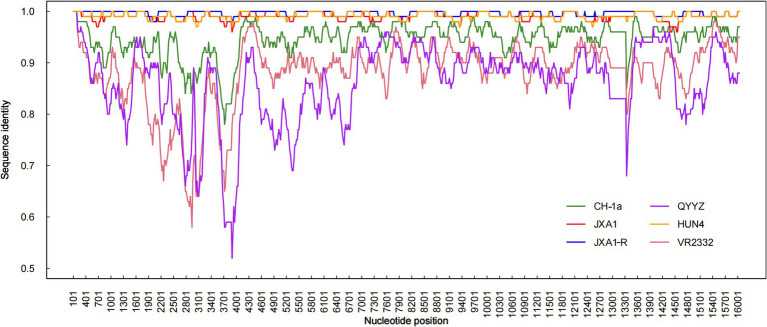
Recombination analysis of NC2023. Similarity plots and bootscan analyses of NC2023 were performed using SimPlot v3.5.1. The complete genome of NC2023 was chosen as the query sequence, and the results were incorporated into R for graphing. The *y*-axis indicates the percentage similarity between the parental sequence and the query sequence.

**Figure 5:**
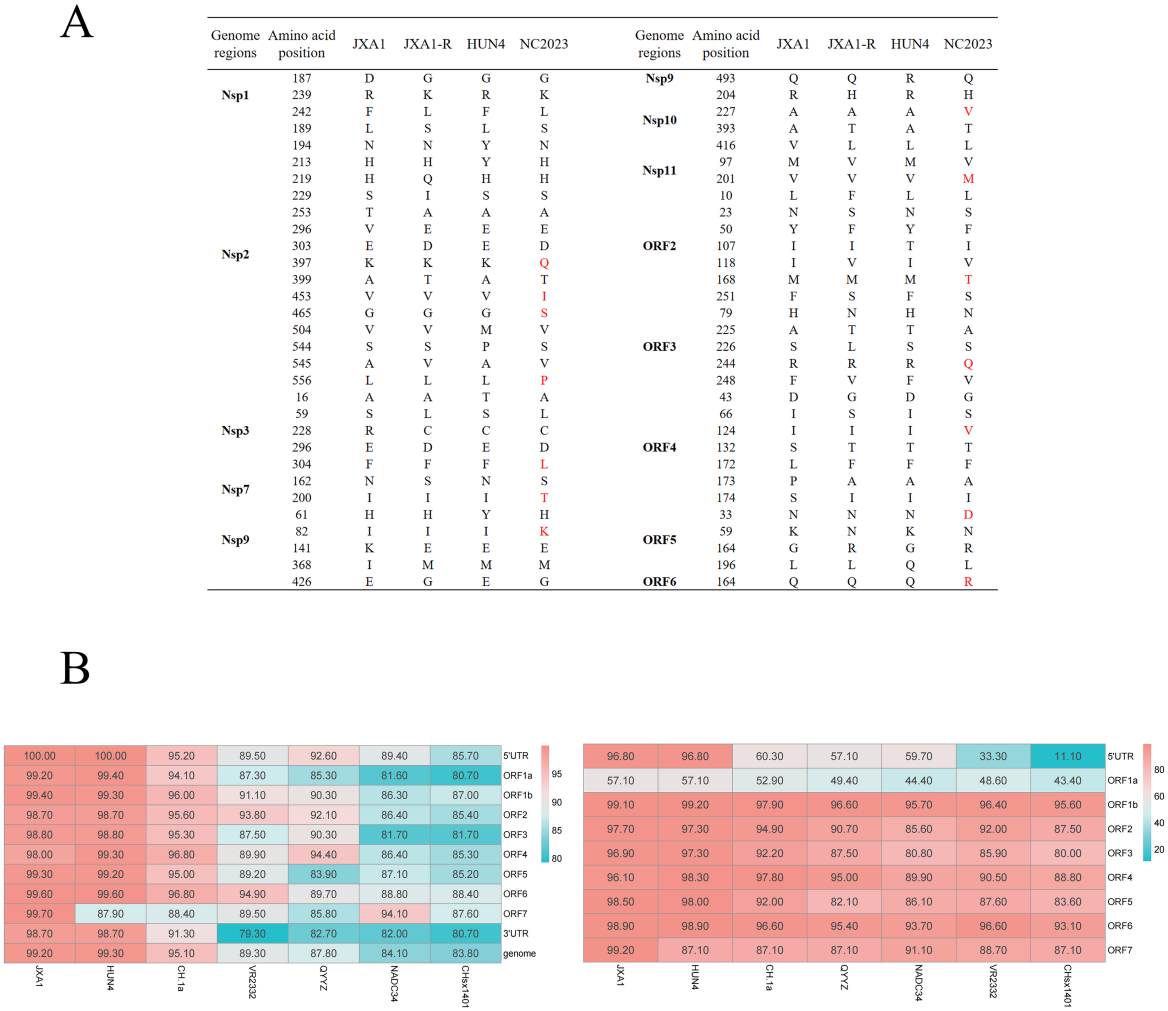
Analysis of sequence insertion and mutation. **(A)** Comparison of NC2023 with recombinant strain in terms of deduced amino acids for each gene. **(B)** Comparison of nucleotide homology (left) and amino acid homology (right) between NC2023 and the classical strain of PRRSV-2.

Recombination analysis performed with SimPlot software, supplemented by phylogenetic visualization in R (Figure 4), revealed minimal recombination signals between NC2023 and lineage 8 strains JXA1, JXA1-R, and HUN4. These results establish NC2023 as a novel PRRSV-2 variant phylogenetically classified within the HP-PRRSV cluster. A comparative analysis of NC2023’s nucleotide and amino acid sequences with those of classical strains was performed, and their similarities were statistically evaluated. As illustrated in Figure 5B, consistent with recombination analysis results, the ORFs of NC2023 show the highest similarity to JXA1 and HUN4, with nucleotide similarities ranging from 83.8 to 99.2% (Left panel). Additionally, the structural proteins of NC2023 display high similarity to various strains, with most mutations occurring in the ORF1a region. To gain a detailed understanding of the variation in NC2023, further analysis of amino acid mutations within each protein was conducted. Comparative analysis of amino acid mutations across viral proteins between NC2023 and the reference strains (JXA1, JXA1-R, and HUN4) revealed distinct substitution patterns in NC2023 (Right panel). Specific amino acid mutation sites and their corresponding altered residues were identified in NC2023 relative to each strain. As shown in Figure 5A, NC2023 exhibited 48, 19, and 47 amino acid differences with JXA1, JXA1-R, and HUN4, respectively. There were 14 unique amino acid mutations in NC2023 that did not overlap with those of the recombinant strains.

### 3.5 Amino acid sequence analysis of Nsp2

**Figure 6:**
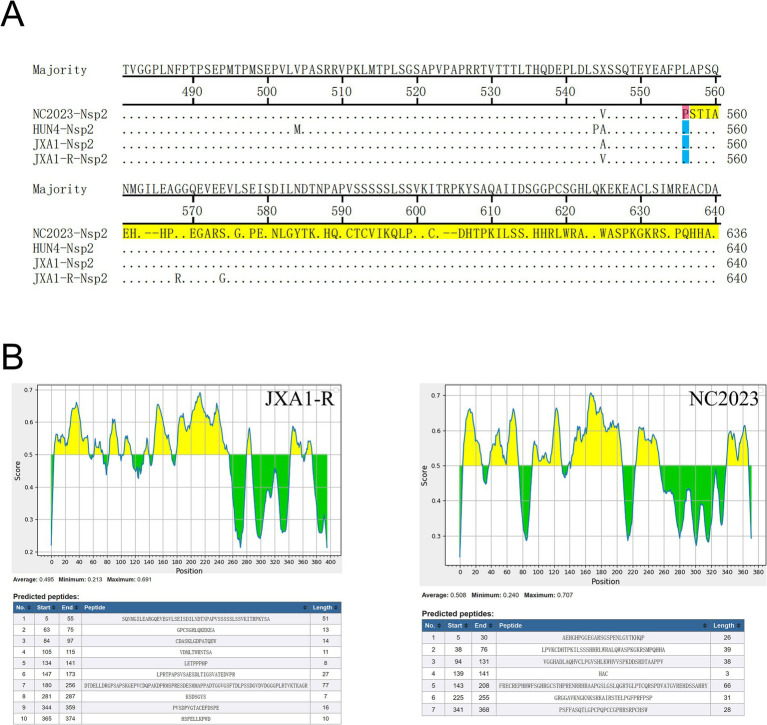
Amino acid sequence analysis of Nsp2. **(A)** Alignment of partial Nsp2 amino acid sequence of NC2023 with HUN4, JXA1, JXA1-R strains. Amino acids that undergo frameshift mutations are displayed with red bars. The amino acids that have undergone significant changes are shown with yellow bars. **(B)** The Nsp2 sequences of JXA1-R and NC2023 starting from the 556th amino acid was predicted by the IEDB; The prediction results of the linear epitopes of the B antigenic epitopes of JXA1-R (left); the prediction results of the linear epitopes of the B antigenic epitopes of NC2023 (right).

Nsp2 is the most variable non-structural protein of PRRSV. Amino acid alignment revealed that the Nsp2 of NC2023 exhibited an insertion of a T base after the 1661st position, resulting in a frameshift mutation affecting 395 amino acids starting from the 556th amino acid (Figure 6A). As shown in Figure 6B, antigenicity prediction was conducted on the sequence following the 556th amino acid in the Nsp2 protein. In JXA1-R, there are 10 predicted antigenic epitopes characterized by a long and concentrated hydrophobic region, with an average antigenic epitope prediction score of 0.483. Conversely, NC2023 has 7 predicted antigenic epitopes with an average prediction score of 0.508. Both the minimum and maximum values have increased, which could affect the antigenicity of the virus and thus influence the infection outcome. These findings indicated that NC2023 is a novel strain and retains viral activity despite significant sequence alterations.

## 4 Discussion

Porcine reproductive and respiratory syndrome virus (PRRSV) remains one of the most significant pathogens affecting the global swine industry, causing substantial economic losses due to reproductive failure in sows and respiratory diseases in growing pigs. The continuous evolution and genetic diversity of PRRSV, particularly through recombination and mutation events, pose significant challenges for disease control and vaccine development (36–38). In this study, we isolated and characterized a novel PRRSV strain, designated NC2023, from a pig farm in Jiangxi Province, China. The results revealed that (i) NC2023 belongs to lineage 8 within the PRRSV-2 subtype and exhibits characteristics of an HP-PRRSV-like strain. (ii) NC2023 is a naturally recombinant strain, having undergone partial recombination with other PRRSV strains from lineage 8, namely JXA1, JXA1-R, and HUN4. (iii) NC2023 carries a frameshift mutation in the Nsp2 gene, without significantly affecting the replication of NC2023 in Marc-145 and iPAM cells.

In 2006, an HP-PRRSV strain lacking 30-amino-acid in the Nsp2 region was first reported, causing a high morbidity rate in pig farms across China. This strain spread rapidly, inflicting substantial economic losses (39). Today, vaccination remains the primary strategy for preventing and controlling PRRS outbreaks in China. Most PRRS vaccines are developed against PRRSV-2 (13). Commercial vaccines including inactivated vaccines, modified live vaccines (MLVs), subunit vaccines, DNA vaccines, and virus-vector vaccines are widely used (40).

Since 2011, MLVs derived from HP-PRRSV strains such as JXA1, HUN4, and TJM have been extensively used in pig farms (41). However, these MLVs provide only partial or no protection against heterologous PRRSV strains (42, 43), and there is a risk of reversion to virulence (44). Additionally, MLV- vaccinated pigs can harbor viremia for up to 4 weeks post-immunization, facilitating transmission of the vaccine virus to unvaccinated pigs (13, 42, 45). This has led to recombination between MLV and wild-type strains, potentially exacerbating the PRRS epidemic (46). Commercially available vaccines in China include VR-2332 (Boehringer-Ingelheim, Mannheim, Germany), CH-1R (DaHuaNong Company, Guangdong, China), JXA1-R (Guangdong Yongshun Biological Pharmaceutical Co. Ltd. Guangdong, China), TJM-F92 (Qingdao Yibang Biological Engineering Co. Ltd) (47). Remarkably, NC2023 shares nearly a identical nucleotide sequence with the commercial vaccine strain JXA1-R, which may be linked to the widespread use of this vaccine in Chinese pig farms. Recombination is a major driver of PRRSV evolution and genetic diversity. The high nucleotide similarity between NC2023 and JXA1-R, particularly in structural protein regions, supports the hypothesis of a recombination event. Although the precise mechanism requires further validation, the genetic data suggest that NC2023 may have acquired selective advantages from both parental strains, potentially contributing to its emergence and spread. Despite this, the delayed appearance of cytopathic effects (CPE) in NC2023-requiring up to 48 h post-plaque purification compared to the 24-h timeline observed with the highly virulent JXA1 strain (Supplementary Figure 3), which may suggest that NC2023 may not exhibit enhanced virulence *in vitro*. Further investigation into its mutation sites could provide insights for attenuating the pathogenicity of vaccines derived from JXA1-R. This study thus lay the groundwork for improving the efficacy and safety of MLV vaccines.

Genomic variations in PRRSV isolates are extensive, particularly in the Nsp2 and ORF5 regions, which exhibit the highest degree of variability. The phenotypic changes in these genes are often indicative of broader genomic alterations (48). Nsp2, in particular, is the largest and most variable non-structural protein in PRRSV. It is commonly subjected to insertions, deletions, recombinations, and amino acid substitutions (49). Nsp2 contains several B-cell epitopes, which are highly immunogenic and capable of inducing antibody production at levels comparable to those of the N protein (50). While both wild-type and attenuated PRRSV strains elicit low cellular immunity, neutralizing antibodies typically appear only in the later stages of infection. Evidence suggests that the protective effect of MLV vaccines is largely mediated by cell-mediated immunity (51). All approved MLV vaccines for PRRS induce weak humoral and cellular immune responses (13). Genetic mutations within the virus contribute to the development of new biological characteristics that alter its antigenicity, enhance pathogenicity, and enable the virus to evade the host immune responses, leading to more severe disease in infected pigs (52, 53). In the NC2023 strain, a frameshift mutation occurs after the 556th amino acid in the Nsp2 protein. *In sillico* epitope predictions using the IEDB online software (threshold set at 0.50) suggested that this mutation may alter the potential antigenic epitope sites, reducing the number from 10 to 7. The mutation also appeared to enhance the hydrophilicity of the peptide segment, suggesting a possible increase in antigenic potential. These computational predictions imply that the Nsp2 mutation might influence the immunogenicity of the virus and could potentially be associated with recent PRRS outbreaks in the region, though further experiments validation is required to confirm these observations.

While our *in vitro* analyses of replication kinetics and phylogenetic relationships provide crucial insights into the potential phenotype and evolutionary history of the NC2023 strain, they cannot fully summarize the complex virus-host interactions that occur *in vivo*. We are unable to definitively characterize key pathogenic traits such as viral virulence, tissue tropism, immunogenicity, and the capacity to cause disease and lesions in a host animal. These *in vivo* properties are ultimately critical for understanding the true threat posed by emerging viral strains and for developing effective control measures. Therefore, future studies involving animal challenge experiments will be indispensable to validate the *in vitro* findings reported here and to fully elucidate the pathogenicity and transmission dynamics of this strain. In conclusion, our study provides valuable insights into the genetic characteristics of PRRSV strain NC2023 and offers a theoretical foundation for improving the efficacy and safety of MLV vaccines. Continued research into the mutation sites of NC2023 may help mitigate its pathogenicity and contribute to better control of PRRS outbreaks.

## Data Availability

The datasets presented in this study can be found in online repositories. The names of the repository/repositories and accession number(s) can be found below: https://www.ncbi.nlm.nih.gov/genbank/, PV342160.
