# Supplementary figures and images for: Isolation and genetic characterization of a novel recombinant HP-PRRSV strain in Jiangxi Province, China
Source: Front Vet Sci. 2025 Oct 6;12:1678378. doi: 10.3389/fvets.2025.1678378 (PMC12536727; doi:10.3389/fvets.2025.1678378)

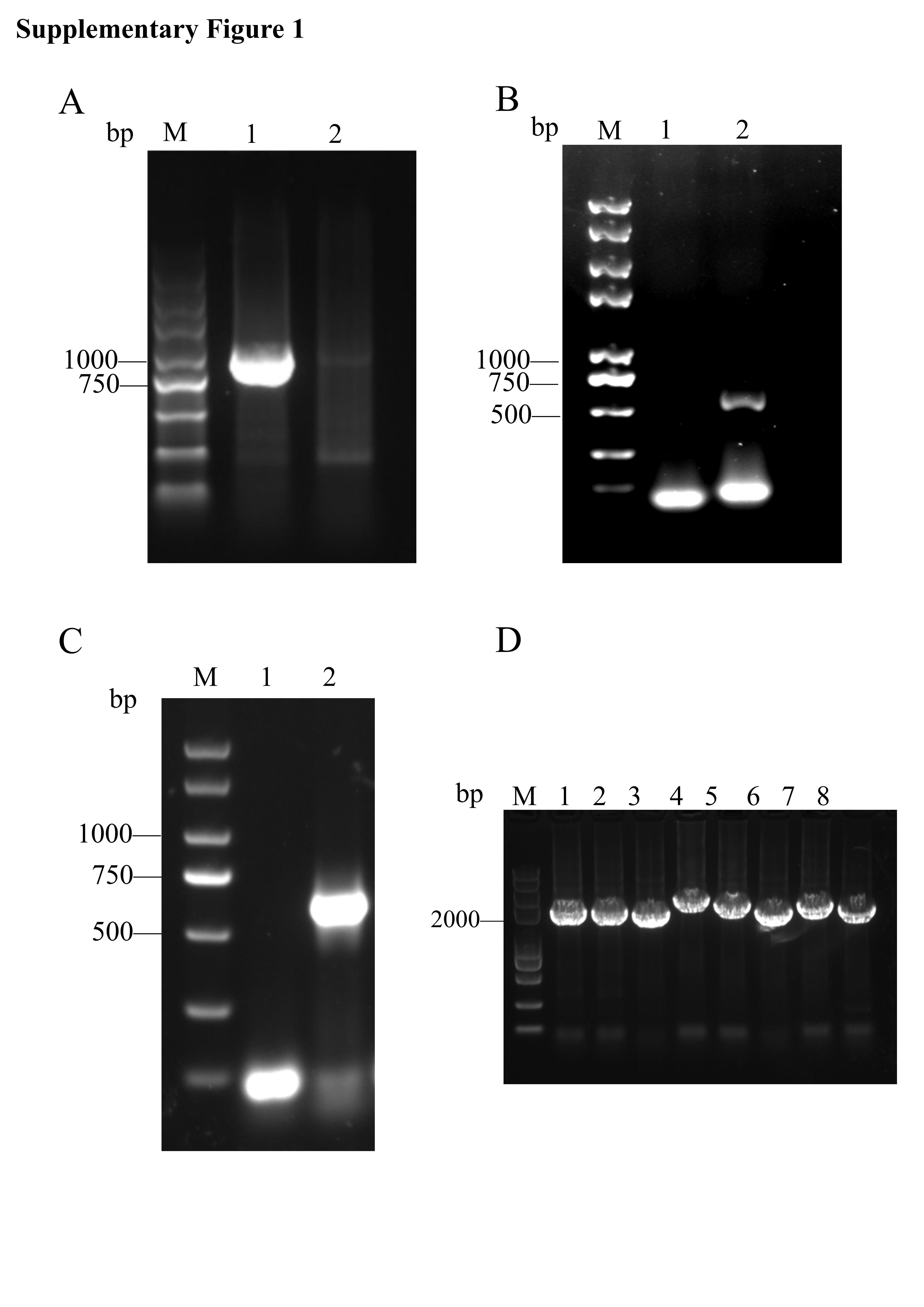

Supplement: Supplementary file 3 [file Image_1.tif]

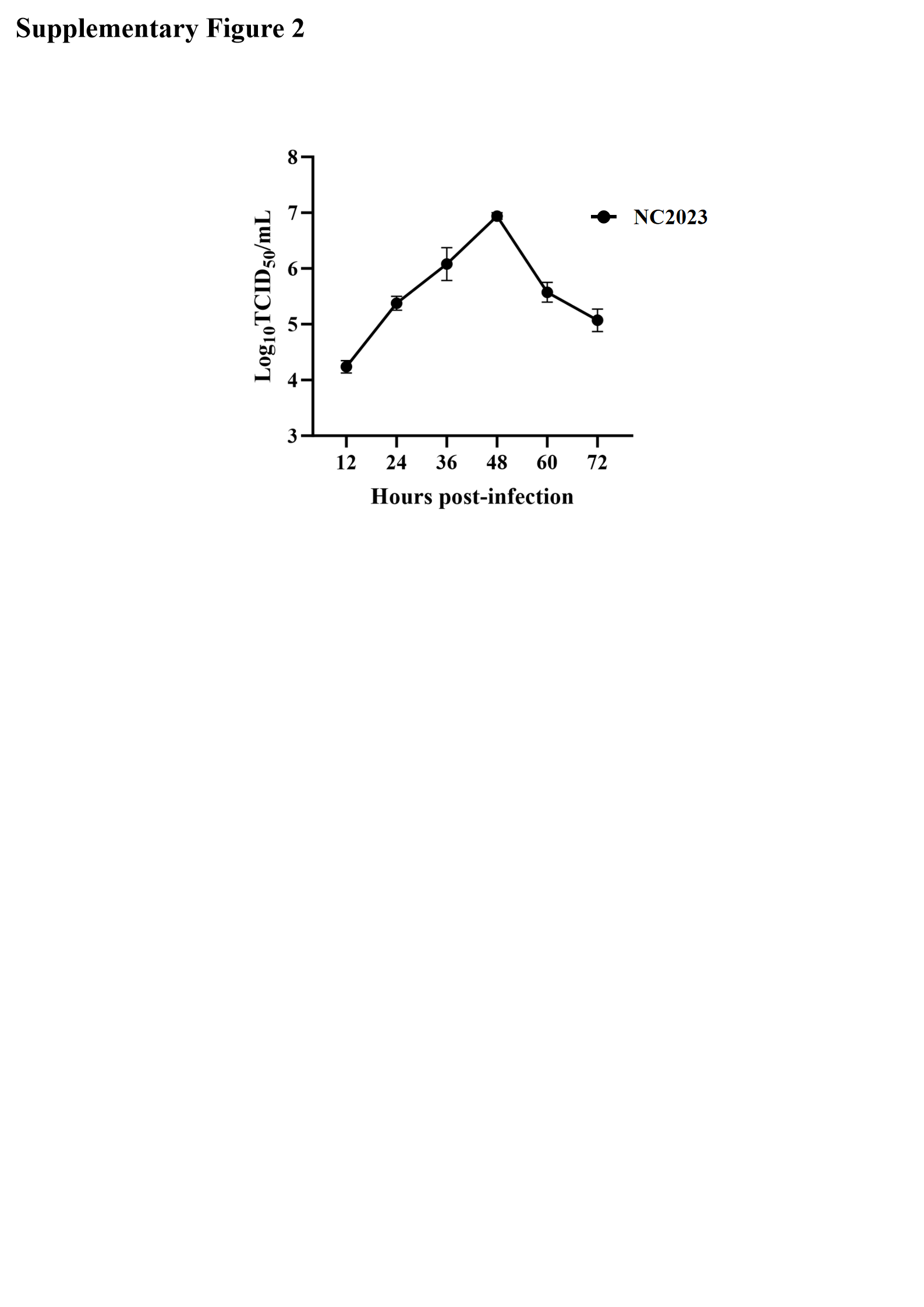

Supplement: Supplementary file 4 [file Image_2.tif]

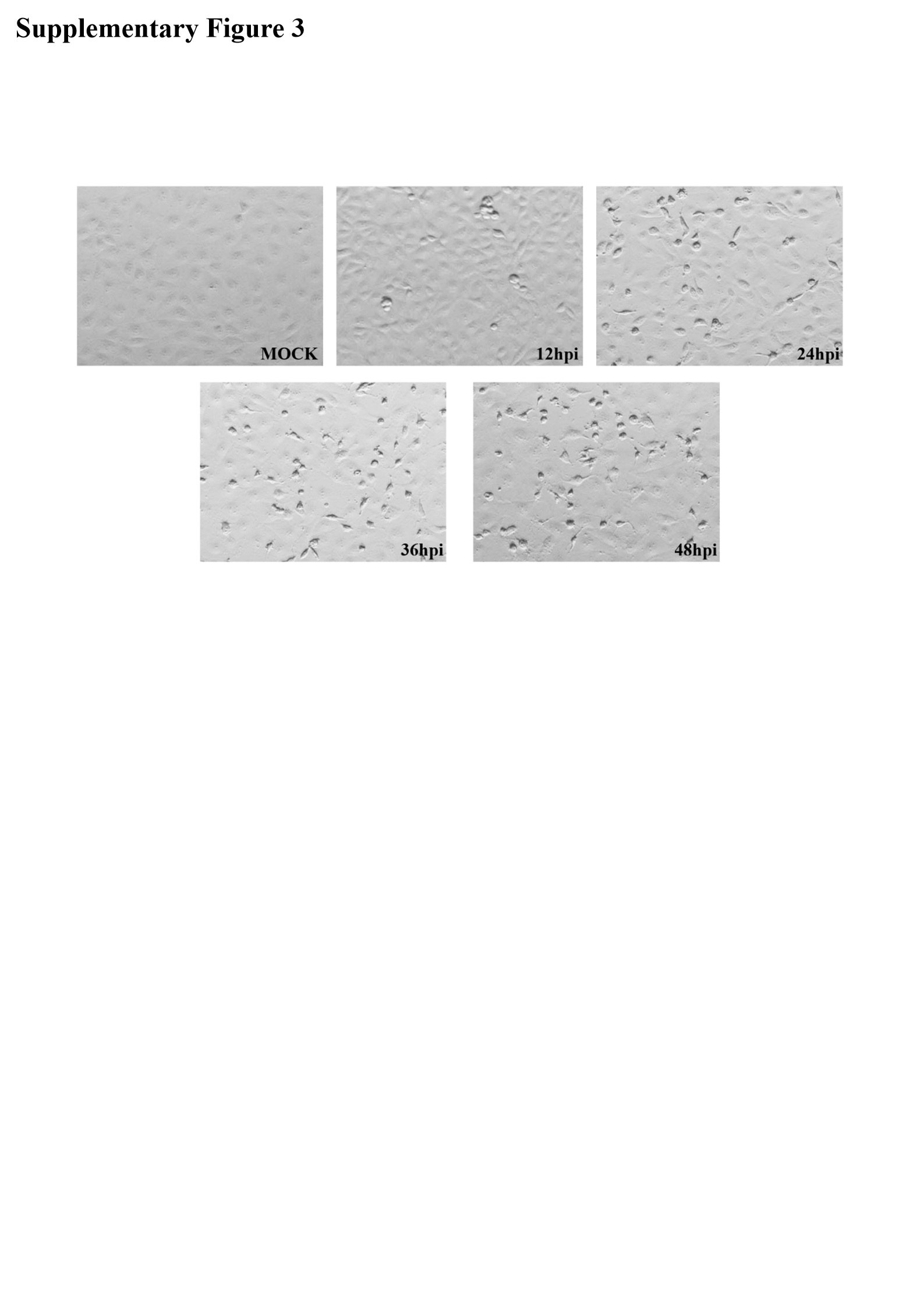

Supplement: Supplementary file 5 [file Image_3.tif]
